# International Medical Congress

**Published:** 1868-02-01

**Authors:** 


### CHICAGO MEDICAL JOURNAL.

*Vol. XXV.—FEBRUARY* 1, 1868.—*No.* 3.

### INTERNATIONAL MEDICAL CONGRESS.*

CONVENED AT PARIS, AUGUST 16, 1867.

TRANSLATED EXPRESSLY FOR THE JOURNAL BY PROF. FREER.

A MALADY may be contagious and virulent, without being transmissible by inoculation under the skin. Inoculate as much as you will the virus of Gonorrhœa, that of contagious Ophthalmia, and you will obtain nothing. Apply them upon a mucous surface, and you will have an affection which offers the same lesions, and which will reproduce the same virus. It is the same with Cholera. M. Roltenkofer, (of Munich), has demonstrated that Cholera is contagious, and that the virus exists in the excrementitious matters. He has cited in its support a large number of facts, collected with care. However, I do not coincide with all the ideas of the Munich school, yet I must admit that they have perfectly demonstrated the point of departure of the contagious principle of Cholera. I have, besides, for my part, observed facts so striking that in their presence it appears to me impossible not to admit the contagiousness of Cholera. The first man attacked with Cholera, who was carried to the Hospital Saint

* Continued from page 18.

Pierre, of Bruxelles, the past year, was placed in a ward by himself, and, as he had no one to watch with him, a robust patient, affected with Chronic Eczema, otherwise perfectly well, to assume this task. This man accepted the task, and passed the night near the patient. The latter recovered; but his guardian contracted the disease, from which he died forty-eight hours after.

In a village two or three leagues from Bruxelles, Cholera did not exist, until a woman came from Bruxelles to visit her daughter; this woman was attacked, and passed it to her child. The doctor of the vicinity — very short sighted, having forgotten his eye glasses — brought the night vessel very close, in order to examine the dejections. Two or three days later, I was summoned by telegraph to render assistance to my confrère. I departed at once; but on my arrival he was already dead of Cholera. At Anderlecht, a village touching Bruxelles, I have been able to follow the march of Cholera from hamlet to hamlet, carried by infected individuals. Besides, experience proves the contagiousness of Cholera. Among animals, the dog is very liable to contract the disease. Cause these animals to swallow the dejections of Cholera, and they will present the usual phenomena of the disease. I have seen one die in twenty-two hours, having had the characteristic discharges, and the usual anatomical lesions of Cholera.

As I have said, it is necessary to take into account, (in the appreciation of the phenomena of contagion), of the matter which is the vehicle, and of the mode of transmission. I do not believe it is transmissible by inoculation, but it is positively by penetration into the buccal and nasal cavities, from the emanations rising from the excreta.

Although I believe in the contagiousness of Cholera, yet I am no partisan of quarantine. I consider it inefficacious, and I do not like the necessary restrictions which are imposed upon commerce and circulation.

Hygienic measures and the immediate disinfection of the dejections, which are the vehicles of the poison, are, according to my belief, the principal prophylactic measures to make use of with advantage.

*M. Marcowitz.*—I would not permit myself to speak upon a subject so vast as Cholera, if I had not been appointed the past year by my Government an inspector of the districts that were being ravaged by this terrible malady, with instructions to make use of all known efficacious measures for combating and preventing the propagation of the disease.

I can not partake of the opinion of M. Shrimpton, upon the non-contagion of Cholera, and without wishing to recall all the proofs which the experience of the past year have furnished, (and of which one may find a large number in the annual dictionary of Dr. Garnier), I may be permitted to remind Dr. Shrimpton that his illustrious compatriot Graves, of Dublin — a long time since — has perfectly established that Cholera has constantly followed in its march the great maritime routes, and had generally appeared in ports where vessels were anchored, from infected places.

The Cholera of the past year has followed, absolutely, the same march. It is thus that it has touched the landings of the lower Rhine, and finished by penetrating the interior of the United Principalities, and following constantly the valleys of the great rivers and the great thoroughfares of communication by land.

A virulent malady may be fatally contagious by inoculation, and eventually contagious by infection. I will not examine here if the contagiousness of infectious maladies in general, and cholera in particular, depends upon the greater intensity of the poison at such and such epochs, or if the poison, being constantly the same cosmic causes, and certain individual conditions can imprint upon it an unaccustomed violence, and render propagation more facile. But that which I will earnestly proclaim is, that cholera demands certain conditions, in order to a great intensity of development. The principal is want and misery. I have constantly observed that the unfortunate portion of the population are those who are almost exclusively struck with the malady. At Bucharest, where the cholera has raged with moderate intensity, it has always been respectful of the class in easy circumstances, and I have constantly left them ignorant of its existence or prevalence, and, at the same time, twenty-five or thirty individuals were dying daily. At Jassy, when I arrived, there were one hundred and fifty dying per day. The medical commission, to which I had the honor of belonging, had already ordained, among other measures, to compel the pauper Jews to remove outside the city, whose slovenliness and disposition to agglomerate rendered them a real focus of infection. This measure, loosely executed, had given insignificant results. With a little energy on my part, and by the aid of much good-will on the part of the Israelitish community, whose sacrifices on this occasion are above all praise, I had succeeded in removing about four thousand, which were lodged in barracks upon the heights of a neighbouring hill, in the best possible condition for perfect æration.

This simple measure had the effect of reducing the mortality to thirty per day. I will not discourse of other measures, such as special hospitals, domiciliary visits, etc., which, incompletely executed, gave but insignificant results.

M. Bonnet, of Bordeaux, regarded quarantines as of great utility, but repelled sanitary *cordons* and all the measures that we might be able to take in order to the prevention of the migration of morbid miasms—miasms in which he did not believe. He counseled, above all, hygienic measures, such as frequent currents of air, the dissemination of the population, and the abandonment of infected districts by all those who can do so without too much inconvenience; the use of *Chlorine*, the disinfection of cess-pools, sobriety, abstinence from fruits green or stale, moderate exercise, a calm spirit, and agreeable distractions, etc.

I have spoken, in order to expose very briefly, how, as a partizan of contagion, when I went to fulfill a mission at the time of the prevalence of cholera in Egypt, I, as well as my colleagues, returned with quite different opinions on this subject. Not but that I believe in contagion, properly so called, that which follows contact, and which one observes in those maladies where the cutaneous secretions are charged with the morbid germs.

I recall that I have made, after many others, an experiment, consisting in enveloping myself, during many nights, in covers that had imbibed the perspiration of those affected with cholera, and that I did not contract the disease. Upon this point, my opinions are well established, and the subject seems to me of great importance, for those attacked with transmissible affections by contact, inspire fright among those who surround them, and are liable to be abandoned, not by the physicians, it is true, but by those who have not assumed the obligations of our calling.

But those who have not this mission, who are not ordinarily accustomed to death-scenes—parents, friends, domestics, all fly. Now, there are not, in either country or city, physicians enough to act in the double capacity of doctor and nurse. When maladies are transmissible otherwise than by contact, the danger seems more distant, and self-love less demonstrative also, the sick are more cared for.

Cholera seems transmissible in this manner. M. Shrimpton has said that a disease, in order to be transmissible, should have a primary period of incubation. Cholera, in effect, has this period. When a vessel puts into an infected port, and if it receives passengers, we often remark that the passengers become choleric many days after embarking. We saw this ourselves on our way to Egypt. The cholera still raged at Malta; we stopped there; a Maltese took passage on our ship, and three days after he died with cholera. Soon other cases declared themselves. We had had seven two days after, when we debarked at Alexandria.

Those who admit that the choleric miasms are carried by the wind, would they contend that in like cases the ship had entered a special current of this kind? But why should the passengers, who came from the city where the epidemic reigned, be the first attacked? It is necessary to admit, in explanation, a period of incubation longer or shorter.

The transmission of cholera by individuals, and above all by sailors, has appeared to us more evident in Egypt, where the disease was not mitigated as in Paris.

Almost always one has been able to verify that the invasion of the malady has coincided with the arrival, either of pilgrims from Mecca, or of travelers who have been subjected to its influences in the countries through which they came.

